# 4-(3-Methoxy­phen­yl)-3-[2-(4-methoxy­phen­yl)eth­yl]-1*H*-1,2,4-triazol-5(4*H*)-one

**DOI:** 10.1107/S1600536809002840

**Published:** 2009-01-28

**Authors:** Muhammad Hanif, Ghulam Qadeer, Nasim Hasan Rama, Javeed Akhtar, Madeleine Helliwell

**Affiliations:** a Department of Chemistry, Quaid-i-Azam University, Islamabad 45320, Pakistan; b The Manchester Materials Science Centre and Department of Chemistry, University of Manchester, Oxford Road, Manchester M13 9PL, England

## Abstract

The asymmetric unit of the title compound, C_18_H_19_N_3_O_3_, contains two crystallographically independent but similar mol­ecules. The triazole ring is oriented with respect to the benzene rings to form dihedral angles of 57.96 (6) and 7.01 (6)° in one mol­ecule, and 64.37 (5) and 10.73 (5)° in the other. The two independent mol­ecules are linked into a dimer by inter­molecular N—H⋯O hydrogen bonds.

## Related literature

For the biological activities of triazole derivatives, see: Demirbas *et al.* (2002 ▶); Holla *et al.* (1998 ▶); Omar *et al.* (1986 ▶); Paulvannan *et al.* (2000 ▶); Turan-Zitouni *et al.* (1999 ▶); Kritsanida *et al.* (2002 ▶). For related structures, see: Öztürk *et al.* (2004*a* ▶,*b* ▶). For hydrogen-bond graph-set terminology, see: Bernstein *et al.* (1995 ▶); Etter (1990 ▶).
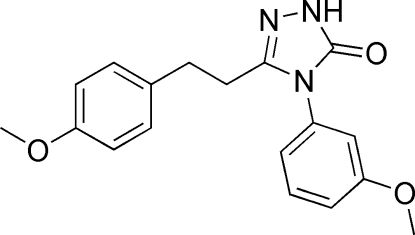


## Experimental

### 

#### Crystal data

C_18_H_19_N_3_O_3_
*M* *_r_* = 325.36
Monoclinic, 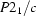

*a* = 26.784 (4) Å
*b* = 14.824 (2) Å
*c* = 8.1108 (11) Å
β = 96.522 (3)°
*V* = 3199.5 (8) Å^3^
*Z* = 8
Mo *K*α radiation
μ = 0.09 mm^−1^
*T* = 100 (2) K
0.50 × 0.50 × 0.20 mm

#### Data collection

Bruker SMART CCD area-detector diffractometer
Absorption correction: none
18196 measured reflections
6524 independent reflections
4463 reflections with *I* > 2σ(*I*)
*R*_int_ = 0.061

#### Refinement

*R*[*F* ^2^ > 2σ(*F* ^2^)] = 0.045
*wR*(*F* ^2^) = 0.083
*S* = 0.93
6524 reflections
445 parameters
H atoms treated by a mixture of independent and constrained refinement
Δρ_max_ = 0.21 e Å^−3^
Δρ_min_ = −0.21 e Å^−3^

### 

Data collection: *SMART* (Bruker, 2001 ▶); cell refinement: *SAINT* (Bruker, 2002 ▶); data reduction: *SAINT*; program(s) used to solve structure: *SHELXS97* (Sheldrick, 2008 ▶); program(s) used to refine structure: *SHELXL97* (Sheldrick, 2008 ▶); molecular graphics: *SHELXTL* (Sheldrick, 2008 ▶); software used to prepare material for publication: *SHELXTL*.

## Supplementary Material

Crystal structure: contains datablocks I, global. DOI: 10.1107/S1600536809002840/rz2291sup1.cif

Structure factors: contains datablocks I. DOI: 10.1107/S1600536809002840/rz2291Isup2.hkl

Additional supplementary materials:  crystallographic information; 3D view; checkCIF report

## Figures and Tables

**Table 1:** Hydrogen-bond geometry (Å, °)

*D*—H⋯*A*	*D*—H	H⋯*A*	*D*⋯*A*	*D*—H⋯*A*
N3—H3N⋯O4^i^	0.972 (18)	1.784 (19)	2.7463 (18)	169.6 (17)
N6—H6N⋯O1^ii^	0.931 (16)	1.936 (17)	2.8429 (18)	164.2 (16)

Symmetry codes: (i) 
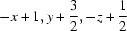
; (ii) 
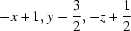
.

**Fig. 1.**
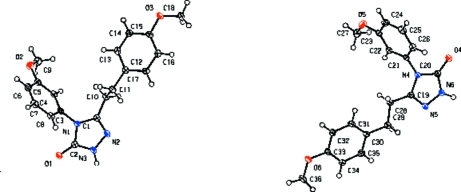
The molecular structure of the title compound with 50% probability displacement ellipsoids (arbitrary spheres for H atoms).

**Fig. 2.**
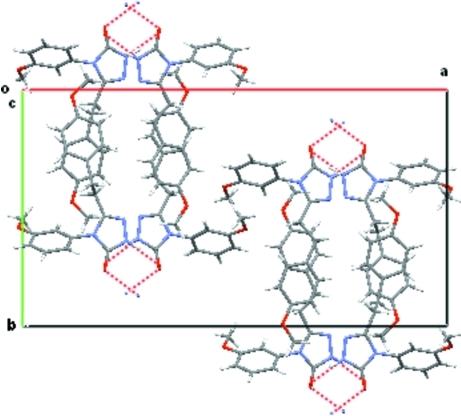
Crystal packing of the title compound viewed along the c axis. Hydrogen bonds are shown as dotted lines.

## supplementary crystallographic information

### Comment

Substituted triazole derivatives are an important class of organic compounds showing significant biological activity, such as antimicrobial (Holla *et al.*, 1998), analgesic (Turan-Zitouni *et al.*, 1999), antitumor (Demirbas *et al.*, 2002), antihypertensive (Paulvannan *et al.*, 2000) and antiviral (Kritsanida *et al.*, 2002) activities. As a continuation of our interest in the synthesis and biological activity of aryloxyacetyl hydrazide derivatives, we report here the synthesis and crystal structure of the title compound (Fig. 1).

The asymmetric unit of the title compound consists of two crystallographically independent molecules with very similar geometry. All bond lengths and angles are unexceptional and comparable with those observed in related structures (Öztürk *et al.*, 2004*a*,*b*). The N2═C1 (1.2955 (19) Å) and N5═C19 (1.2971 (19) Å) bonds show double bond character. The dihedral angles formed by the triazole ring with the aromatic rings of the 4-methoxyphenyl and 3-methoxyphenyl groups are 57.96 (6) and 7.01 (6)° in the molecule containing the N1–N3 atoms, and 64.37 (6) and 10.73 (5)° in the molecule containing the N4–N6 atoms. In the crystal packing (Fig. 2), the two independent molecules are linked into a dimer by intermolecular N—H···O hydrogen bonds (Table 1) generating a ring of graph-set *R*^2^_2_(8) (Etter, 1990; Bernstein *et al.*,  1995).

### Experimental

The synthesis of the title compound was carried out by refluxing a solution of 4-(3-methoxyphenyl)-1-(3-(4-methoxyphenyl)propanoyl)semicarbazide (3.43 g, 10 mmol) in 2 *M* NaOH for 5 h. Single crystals suitable for X-ray measurements were obtained on slow evaporation of an aqeous ethanol solution at room temperature (yield: 90%; m.p. 407–408 K).

### Refinement

H atoms bonded to C atoms were included in calculated positions and refined using the riding model approximation, with C—H = 0.95-0.99 Å and with *U*_iso_(H) = 1.2 *U*_eq_(C) or  1.5 *U*_eq_(C) for methyl H atoms. Atoms H3N and H6N were located in a difference Fourier map and refined isotropically.

### Crystal data

**Table:**

C_18_H_19_N_3_O_3_	*F*(000) = 1376
*M**_r_* = 325.36	*D*_x_ = 1.351 Mg m^−^^3^
Monoclinic, *P*2_1_/*c*	Melting point: 407(1) K
Hall symbol: -P 2ybc	Mo *K*α radiation, λ = 0.71073 Å
*a* = 26.784 (4) Å	Cell parameters from 1009 reflections
*b* = 14.824 (2) Å	θ = 2.7–26.4°
*c* = 8.1108 (11) Å	µ = 0.09 mm^−^^1^
β = 96.522 (3)°	*T* = 100 K
*V* = 3199.5 (8) Å^3^	Irregular, colourless
*Z* = 8	0.50 × 0.50 × 0.20 mm

### Data collection

**Table:**

Bruker SMART CCD area-detector diffractometer	4463 reflections with *I* > 2σ(*I*)
Radiation source: fine-focus sealed tube	*R*_int_ = 0.061
graphite	θ_max_ = 26.4°, θ_min_ = 2.1°
φ and ω scans	*h* = −29→33
18196 measured reflections	*k* = −9→18
6524 independent reflections	*l* = −10→9

### Refinement

**Table:**

Refinement on *F*^2^	Primary atom site location: structure-invariant direct methods
Least-squares matrix: full	Secondary atom site location: difference Fourier map
*R*[*F*^2^ > 2σ(*F*^2^)] = 0.045	Hydrogen site location: inferred from neighbouring sites
*wR*(*F*^2^) = 0.083	H atoms treated by a mixture of independent and constrained refinement
*S* = 0.93	*w* = 1/[σ^2^(*F*_o_^2^) + (0.022*P*)^2^] where *P* = (*F*_o_^2^ + 2*F*_c_^2^)/3
6524 reflections	(Δ/σ)_max_ = 0.001
445 parameters	Δρ_max_ = 0.21 e Å^−^^3^
0 restraints	Δρ_min_ = −0.21 e Å^−^^3^

### Special details

**Table:**

Geometry. All e.s.d.'s (except the e.s.d. in the dihedral angle between two l.s. planes) are estimated using the full covariance matrix. The cell e.s.d.'s are taken into account individually in the estimation of e.s.d.'s in distances, angles and torsion angles; correlations between e.s.d.'s in cell parameters are only used when they are defined by crystal symmetry. An approximate (isotropic) treatment of cell e.s.d.'s is used for estimating e.s.d.'s involving l.s. planes.
Refinement. Refinement of *F*^2^ against ALL reflections. The weighted *R*-factor *wR* and goodness of fit *S* are based on *F*^2^, conventional *R*-factors *R* are based on *F*, with *F* set to zero for negative *F*^2^. The threshold expression of *F*^2^ > σ(*F*^2^) is used only for calculating *R*-factors(gt) *etc*. and is not relevant to the choice of reflections for refinement. *R*-factors based on *F*^2^ are statistically about twice as large as those based on *F*, and *R*- factors based on ALL data will be even larger.

### Fractional atomic coordinates and isotropic or equivalent isotropic displacement parameters (Å^2^)

**Table:**

	*x*	*y*	*z*	*U*_iso_*/*U*_eq_	
O1	0.80652 (4)	1.26410 (8)	0.38630 (14)	0.0241 (3)	
O2	1.01067 (4)	1.08446 (8)	0.35402 (14)	0.0250 (3)	
O3	0.88400 (4)	0.52515 (8)	0.49269 (14)	0.0224 (3)	
N1	0.83241 (5)	1.11201 (9)	0.40707 (16)	0.0168 (3)	
N2	0.76150 (5)	1.04721 (9)	0.30095 (16)	0.0200 (3)	
N3	0.75591 (5)	1.14005 (10)	0.30968 (18)	0.0210 (3)	
H3N	0.7259 (6)	1.1699 (13)	0.258 (2)	0.049 (6)*	
C1	0.80770 (6)	1.03201 (11)	0.36030 (19)	0.0165 (4)	
C2	0.79850 (6)	1.18218 (12)	0.3694 (2)	0.0196 (4)	
C3	0.88409 (6)	1.12582 (11)	0.4688 (2)	0.0170 (4)	
C4	0.92115 (6)	1.09601 (11)	0.37531 (19)	0.0176 (4)	
H4	0.9124	1.0662	0.2724	0.021*	
C5	0.97099 (6)	1.11055 (11)	0.4347 (2)	0.0188 (4)	
C6	0.98332 (6)	1.15633 (12)	0.5836 (2)	0.0229 (4)	
H6	1.0176	1.1670	0.6233	0.027*	
C7	0.94588 (6)	1.18619 (12)	0.6732 (2)	0.0232 (4)	
H7	0.9546	1.2175	0.7746	0.028*	
C8	0.89570 (6)	1.17121 (11)	0.6175 (2)	0.0208 (4)	
H8	0.8699	1.1916	0.6798	0.025*	
C9	1.00001 (6)	1.04758 (13)	0.1916 (2)	0.0273 (5)	
H9A	0.9791	0.9936	0.1962	0.041*	
H9B	1.0316	1.0314	0.1485	0.041*	
H9C	0.9821	1.0924	0.1184	0.041*	
C10	0.82961 (6)	0.93970 (11)	0.37288 (19)	0.0184 (4)	
H10A	0.8628	0.9416	0.3299	0.022*	
H10B	0.8077	0.8993	0.2991	0.022*	
C11	0.83669 (6)	0.89770 (11)	0.54600 (19)	0.0199 (4)	
H11A	0.8646	0.9285	0.6145	0.024*	
H11B	0.8057	0.9059	0.6002	0.024*	
C12	0.84841 (6)	0.79782 (12)	0.53503 (19)	0.0166 (4)	
C13	0.89660 (6)	0.76489 (12)	0.57477 (19)	0.0199 (4)	
H13	0.9228	0.8051	0.6154	0.024*	
C14	0.90727 (6)	0.67427 (12)	0.5562 (2)	0.0204 (4)	
H14	0.9407	0.6532	0.5820	0.024*	
C15	0.86938 (6)	0.61408 (11)	0.50016 (19)	0.0169 (4)	
C16	0.82093 (6)	0.64531 (12)	0.46004 (19)	0.0178 (4)	
H16	0.7947	0.6049	0.4210	0.021*	
C17	0.81113 (6)	0.73673 (12)	0.47759 (19)	0.0189 (4)	
H17	0.7778	0.7581	0.4493	0.023*	
C18	0.84577 (6)	0.45979 (12)	0.4460 (2)	0.0233 (4)	
H18A	0.8198	0.4635	0.5218	0.035*	
H18B	0.8606	0.3993	0.4518	0.035*	
H18C	0.8307	0.4718	0.3323	0.035*	
O4	0.32218 (4)	−0.25770 (8)	0.33832 (14)	0.0232 (3)	
O5	0.52563 (4)	−0.08829 (9)	0.35036 (14)	0.0252 (3)	
O6	0.38708 (4)	0.49050 (8)	0.53429 (13)	0.0214 (3)	
N4	0.34705 (5)	−0.10875 (9)	0.40056 (16)	0.0167 (3)	
N5	0.27715 (5)	−0.03721 (9)	0.30255 (17)	0.0200 (3)	
N6	0.27211 (5)	−0.13024 (9)	0.28110 (18)	0.0198 (3)	
H6N	0.2432 (6)	−0.1546 (12)	0.223 (2)	0.034 (5)*	
C19	0.32257 (6)	−0.02667 (11)	0.3744 (2)	0.0173 (4)	
C20	0.31384 (6)	−0.17568 (12)	0.3389 (2)	0.0182 (4)	
C21	0.39848 (6)	−0.12574 (11)	0.4633 (2)	0.0161 (4)	
C22	0.43614 (6)	−0.09321 (11)	0.37456 (19)	0.0168 (4)	
H22	0.4281	−0.0577	0.2777	0.020*	
C23	0.48550 (6)	−0.11357 (11)	0.4300 (2)	0.0177 (4)	
C24	0.49701 (6)	−0.16566 (12)	0.5723 (2)	0.0219 (4)	
H24	0.5310	−0.1801	0.6096	0.026*	
C25	0.45893 (6)	−0.19599 (12)	0.6587 (2)	0.0234 (4)	
H25	0.4670	−0.2306	0.7567	0.028*	
C26	0.40919 (6)	−0.17697 (12)	0.6052 (2)	0.0210 (4)	
H26	0.3830	−0.1986	0.6646	0.025*	
C27	0.51482 (6)	−0.04444 (13)	0.1937 (2)	0.0276 (4)	
H27A	0.4942	−0.0842	0.1171	0.041*	
H27B	0.5463	−0.0306	0.1483	0.041*	
H27C	0.4965	0.0117	0.2082	0.041*	
C28	0.34597 (6)	0.06097 (11)	0.4281 (2)	0.0192 (4)	
H28A	0.3745	0.0731	0.3635	0.023*	
H28B	0.3594	0.0571	0.5467	0.023*	
C29	0.30904 (6)	0.13794 (11)	0.4045 (2)	0.0253 (4)	
H29A	0.2825	0.1279	0.4782	0.030*	
H29B	0.2927	0.1363	0.2887	0.030*	
C30	0.33070 (6)	0.23100 (11)	0.4386 (2)	0.0179 (4)	
C31	0.37623 (6)	0.24682 (12)	0.5333 (2)	0.0201 (4)	
H31	0.3953	0.1972	0.5803	0.024*	
C32	0.39445 (6)	0.33361 (11)	0.5607 (2)	0.0195 (4)	
H32	0.4262	0.3431	0.6233	0.023*	
C33	0.36632 (6)	0.40670 (11)	0.49654 (19)	0.0166 (4)	
C34	0.32082 (6)	0.39336 (12)	0.40142 (19)	0.0182 (4)	
H34	0.3015	0.4430	0.3559	0.022*	
C35	0.30397 (6)	0.30552 (12)	0.3740 (2)	0.0199 (4)	
H35	0.2728	0.2960	0.3080	0.024*	
C36	0.35951 (6)	0.56700 (11)	0.4650 (2)	0.0235 (4)	
H36A	0.3559	0.5629	0.3436	0.035*	
H36B	0.3776	0.6224	0.5001	0.035*	
H36C	0.3262	0.5680	0.5039	0.035*	

### Atomic displacement parameters (Å^2^)

**Table:**

	*U*^11^	*U*^22^	*U*^33^	*U*^12^	*U*^13^	*U*^23^
O1	0.0235 (7)	0.0120 (7)	0.0356 (8)	−0.0005 (6)	−0.0020 (5)	0.0000 (6)
O2	0.0208 (6)	0.0277 (8)	0.0267 (7)	−0.0027 (6)	0.0036 (5)	−0.0061 (6)
O3	0.0244 (7)	0.0133 (7)	0.0288 (7)	0.0011 (6)	0.0003 (5)	−0.0011 (5)
N1	0.0169 (7)	0.0131 (8)	0.0200 (8)	0.0007 (6)	0.0000 (6)	0.0004 (6)
N2	0.0230 (8)	0.0126 (8)	0.0241 (8)	−0.0008 (7)	0.0020 (6)	−0.0007 (6)
N3	0.0212 (8)	0.0126 (8)	0.0285 (9)	0.0008 (7)	−0.0008 (7)	0.0017 (7)
C1	0.0200 (9)	0.0159 (10)	0.0138 (9)	−0.0008 (8)	0.0031 (7)	0.0000 (7)
C2	0.0206 (10)	0.0177 (10)	0.0205 (10)	−0.0011 (8)	0.0024 (7)	0.0003 (8)
C3	0.0187 (9)	0.0120 (9)	0.0196 (9)	−0.0007 (8)	−0.0008 (7)	0.0037 (7)
C4	0.0243 (10)	0.0133 (9)	0.0148 (9)	−0.0020 (8)	0.0001 (7)	0.0013 (7)
C5	0.0222 (9)	0.0148 (10)	0.0191 (9)	−0.0005 (8)	0.0006 (7)	0.0013 (8)
C6	0.0218 (9)	0.0200 (10)	0.0253 (10)	−0.0024 (8)	−0.0040 (8)	0.0010 (8)
C7	0.0293 (10)	0.0204 (11)	0.0183 (10)	0.0000 (9)	−0.0046 (8)	−0.0036 (8)
C8	0.0269 (10)	0.0151 (10)	0.0205 (10)	0.0009 (8)	0.0038 (8)	0.0003 (8)
C9	0.0265 (10)	0.0282 (12)	0.0276 (11)	−0.0013 (9)	0.0058 (8)	−0.0073 (9)
C10	0.0220 (9)	0.0150 (10)	0.0182 (9)	−0.0002 (8)	0.0027 (7)	−0.0005 (7)
C11	0.0228 (9)	0.0180 (10)	0.0188 (9)	0.0000 (8)	0.0023 (7)	−0.0017 (8)
C12	0.0221 (9)	0.0171 (10)	0.0112 (9)	−0.0003 (8)	0.0038 (7)	0.0010 (7)
C13	0.0225 (10)	0.0181 (10)	0.0191 (10)	−0.0044 (8)	0.0020 (7)	−0.0010 (8)
C14	0.0195 (9)	0.0193 (10)	0.0218 (10)	0.0014 (8)	0.0002 (7)	0.0008 (8)
C15	0.0240 (9)	0.0148 (10)	0.0122 (9)	0.0025 (8)	0.0035 (7)	0.0020 (7)
C16	0.0201 (9)	0.0194 (10)	0.0137 (9)	−0.0035 (8)	0.0006 (7)	−0.0005 (7)
C17	0.0204 (9)	0.0203 (10)	0.0162 (9)	0.0044 (8)	0.0026 (7)	0.0039 (8)
C18	0.0307 (10)	0.0154 (10)	0.0236 (10)	−0.0050 (9)	0.0025 (8)	−0.0023 (8)
O4	0.0222 (6)	0.0125 (7)	0.0346 (7)	0.0001 (6)	0.0016 (5)	−0.0010 (6)
O5	0.0186 (6)	0.0301 (8)	0.0274 (7)	−0.0001 (6)	0.0044 (5)	0.0034 (6)
O6	0.0244 (6)	0.0121 (7)	0.0264 (7)	0.0008 (5)	−0.0024 (5)	0.0000 (5)
N4	0.0168 (7)	0.0117 (8)	0.0219 (8)	0.0002 (6)	0.0029 (6)	−0.0008 (6)
N5	0.0220 (8)	0.0119 (8)	0.0263 (8)	−0.0005 (7)	0.0037 (6)	−0.0028 (7)
N6	0.0172 (8)	0.0130 (8)	0.0283 (9)	−0.0007 (7)	−0.0002 (7)	−0.0035 (7)
C19	0.0174 (9)	0.0148 (10)	0.0203 (9)	0.0018 (8)	0.0048 (7)	−0.0002 (8)
C20	0.0191 (9)	0.0168 (10)	0.0194 (9)	−0.0016 (8)	0.0056 (7)	−0.0016 (8)
C21	0.0171 (9)	0.0116 (9)	0.0193 (9)	−0.0007 (7)	0.0009 (7)	−0.0047 (7)
C22	0.0216 (9)	0.0127 (9)	0.0157 (9)	0.0013 (8)	0.0004 (7)	−0.0001 (7)
C23	0.0186 (9)	0.0139 (9)	0.0206 (9)	−0.0007 (8)	0.0027 (7)	−0.0051 (7)
C24	0.0207 (9)	0.0201 (10)	0.0234 (10)	0.0048 (8)	−0.0047 (8)	−0.0021 (8)
C25	0.0331 (11)	0.0190 (10)	0.0169 (9)	0.0008 (9)	−0.0017 (8)	0.0025 (8)
C26	0.0256 (10)	0.0181 (10)	0.0194 (10)	−0.0022 (8)	0.0038 (8)	−0.0005 (8)
C27	0.0273 (10)	0.0301 (12)	0.0270 (11)	−0.0023 (9)	0.0095 (8)	0.0062 (9)
C28	0.0198 (9)	0.0160 (10)	0.0216 (10)	−0.0002 (8)	0.0012 (7)	−0.0010 (8)
C29	0.0195 (9)	0.0176 (11)	0.0381 (11)	0.0006 (8)	0.0002 (8)	−0.0051 (9)
C30	0.0181 (9)	0.0142 (10)	0.0217 (10)	0.0013 (8)	0.0037 (7)	−0.0028 (8)
C31	0.0216 (9)	0.0147 (10)	0.0236 (10)	0.0038 (8)	0.0014 (7)	0.0017 (8)
C32	0.0188 (9)	0.0174 (10)	0.0212 (10)	−0.0010 (8)	−0.0023 (7)	−0.0004 (8)
C33	0.0204 (9)	0.0137 (9)	0.0161 (9)	−0.0033 (8)	0.0043 (7)	−0.0033 (7)
C34	0.0196 (9)	0.0154 (10)	0.0193 (9)	0.0041 (8)	0.0009 (7)	0.0017 (8)
C35	0.0174 (9)	0.0224 (11)	0.0192 (10)	−0.0004 (8)	−0.0009 (7)	−0.0016 (8)
C36	0.0317 (10)	0.0122 (10)	0.0260 (10)	0.0016 (8)	0.0005 (8)	0.0015 (8)

### Geometric parameters (Å, °)

**Table:**

O1—C2	1.238 (2)	O4—C20	1.236 (2)
O2—C5	1.3656 (18)	O5—C23	1.3677 (18)
O2—C9	1.4248 (18)	O5—C27	1.4272 (19)
O3—C15	1.3786 (19)	O6—C33	1.3810 (19)
O3—C18	1.4292 (18)	O6—C36	1.4328 (19)
N1—C1	1.390 (2)	N4—C19	1.387 (2)
N1—C2	1.392 (2)	N4—C20	1.387 (2)
N1—C3	1.4323 (19)	N4—C21	1.4354 (19)
N2—C1	1.2955 (19)	N5—C19	1.2971 (19)
N2—N3	1.3871 (19)	N5—N6	1.3947 (18)
N3—C2	1.342 (2)	N6—C20	1.343 (2)
N3—H3N	0.972 (18)	N6—H6N	0.931 (16)
C1—C10	1.488 (2)	C19—C28	1.486 (2)
C3—C8	1.385 (2)	C21—C26	1.382 (2)
C3—C4	1.388 (2)	C21—C22	1.390 (2)
C4—C5	1.384 (2)	C22—C23	1.381 (2)
C4—H4	0.9500	C22—H22	0.9500
C5—C6	1.392 (2)	C23—C24	1.394 (2)
C6—C7	1.377 (2)	C24—C25	1.377 (2)
C6—H6	0.9500	C24—H24	0.9500
C7—C8	1.386 (2)	C25—C26	1.383 (2)
C7—H7	0.9500	C25—H25	0.9500
C8—H8	0.9500	C26—H26	0.9500
C9—H9A	0.9800	C27—H27A	0.9800
C9—H9B	0.9800	C27—H27B	0.9800
C9—H9C	0.9800	C27—H27C	0.9800
C10—C11	1.528 (2)	C28—C29	1.508 (2)
C10—H10A	0.9900	C28—H28A	0.9900
C10—H10B	0.9900	C28—H28B	0.9900
C11—C12	1.518 (2)	C29—C30	1.510 (2)
C11—H11A	0.9900	C29—H29A	0.9900
C11—H11B	0.9900	C29—H29B	0.9900
C12—C13	1.383 (2)	C30—C31	1.385 (2)
C12—C17	1.389 (2)	C30—C35	1.386 (2)
C13—C14	1.385 (2)	C31—C32	1.385 (2)
C13—H13	0.9500	C31—H31	0.9500
C14—C15	1.388 (2)	C32—C33	1.387 (2)
C14—H14	0.9500	C32—H32	0.9500
C15—C16	1.381 (2)	C33—C34	1.381 (2)
C16—C17	1.391 (2)	C34—C35	1.388 (2)
C16—H16	0.9500	C34—H34	0.9500
C17—H17	0.9500	C35—H35	0.9500
C18—H18A	0.9800	C36—H36A	0.9800
C18—H18B	0.9800	C36—H36B	0.9800
C18—H18C	0.9800	C36—H36C	0.9800
			
C5—O2—C9	117.80 (12)	C23—O5—C27	117.02 (12)
C15—O3—C18	117.49 (12)	C33—O6—C36	116.64 (12)
C1—N1—C2	107.42 (13)	C19—N4—C20	107.44 (13)
C1—N1—C3	128.95 (14)	C19—N4—C21	128.77 (14)
C2—N1—C3	123.41 (14)	C20—N4—C21	123.53 (14)
C1—N2—N3	104.94 (14)	C19—N5—N6	104.34 (13)
C2—N3—N2	112.82 (14)	C20—N6—N5	112.79 (13)
C2—N3—H3N	124.9 (11)	C20—N6—H6N	126.3 (11)
N2—N3—H3N	121.2 (11)	N5—N6—H6N	120.7 (11)
N2—C1—N1	110.98 (15)	N5—C19—N4	111.47 (15)
N2—C1—C10	122.56 (15)	N5—C19—C28	125.42 (15)
N1—C1—C10	126.46 (14)	N4—C19—C28	123.08 (14)
O1—C2—N3	128.76 (16)	O4—C20—N6	129.45 (16)
O1—C2—N1	127.46 (15)	O4—C20—N4	126.59 (15)
N3—C2—N1	103.78 (14)	N6—C20—N4	103.95 (14)
C8—C3—C4	121.78 (15)	C26—C21—C22	121.84 (15)
C8—C3—N1	119.09 (14)	C26—C21—N4	119.46 (15)
C4—C3—N1	119.09 (14)	C22—C21—N4	118.64 (14)
C5—C4—C3	118.80 (15)	C23—C22—C21	118.69 (15)
C5—C4—H4	120.6	C23—C22—H22	120.7
C3—C4—H4	120.6	C21—C22—H22	120.7
O2—C5—C4	124.17 (15)	O5—C23—C22	124.15 (15)
O2—C5—C6	115.64 (14)	O5—C23—C24	115.53 (14)
C4—C5—C6	120.16 (16)	C22—C23—C24	120.28 (15)
C7—C6—C5	119.97 (16)	C25—C24—C23	119.69 (15)
C7—C6—H6	120.0	C25—C24—H24	120.2
C5—C6—H6	120.0	C23—C24—H24	120.2
C6—C7—C8	120.93 (16)	C24—C25—C26	121.12 (16)
C6—C7—H7	119.5	C24—C25—H25	119.4
C8—C7—H7	119.5	C26—C25—H25	119.4
C3—C8—C7	118.34 (16)	C25—C26—C21	118.37 (16)
C3—C8—H8	120.8	C25—C26—H26	120.8
C7—C8—H8	120.8	C21—C26—H26	120.8
O2—C9—H9A	109.5	O5—C27—H27A	109.5
O2—C9—H9B	109.5	O5—C27—H27B	109.5
H9A—C9—H9B	109.5	H27A—C27—H27B	109.5
O2—C9—H9C	109.5	O5—C27—H27C	109.5
H9A—C9—H9C	109.5	H27A—C27—H27C	109.5
H9B—C9—H9C	109.5	H27B—C27—H27C	109.5
C1—C10—C11	116.39 (14)	C19—C28—C29	112.07 (13)
C1—C10—H10A	108.2	C19—C28—H28A	109.2
C11—C10—H10A	108.2	C29—C28—H28A	109.2
C1—C10—H10B	108.2	C19—C28—H28B	109.2
C11—C10—H10B	108.2	C29—C28—H28B	109.2
H10A—C10—H10B	107.3	H28A—C28—H28B	107.9
C12—C11—C10	110.39 (13)	C28—C29—C30	115.81 (13)
C12—C11—H11A	109.6	C28—C29—H29A	108.3
C10—C11—H11A	109.6	C30—C29—H29A	108.3
C12—C11—H11B	109.6	C28—C29—H29B	108.3
C10—C11—H11B	109.6	C30—C29—H29B	108.3
H11A—C11—H11B	108.1	H29A—C29—H29B	107.4
C13—C12—C17	117.66 (16)	C31—C30—C35	117.32 (16)
C13—C12—C11	121.60 (15)	C31—C30—C29	123.49 (15)
C17—C12—C11	120.68 (14)	C35—C30—C29	119.19 (14)
C12—C13—C14	121.03 (16)	C30—C31—C32	121.21 (16)
C12—C13—H13	119.5	C30—C31—H31	119.4
C14—C13—H13	119.5	C32—C31—H31	119.4
C13—C14—C15	120.43 (15)	C31—C32—C33	119.94 (15)
C13—C14—H14	119.8	C31—C32—H32	120.0
C15—C14—H14	119.8	C33—C32—H32	120.0
O3—C15—C16	125.02 (15)	C34—C33—O6	124.08 (15)
O3—C15—C14	115.36 (14)	C34—C33—C32	120.34 (16)
C16—C15—C14	119.60 (16)	O6—C33—C32	115.58 (14)
C15—C16—C17	119.05 (16)	C33—C34—C35	118.32 (16)
C15—C16—H16	120.5	C33—C34—H34	120.8
C17—C16—H16	120.5	C35—C34—H34	120.8
C12—C17—C16	122.20 (15)	C30—C35—C34	122.85 (15)
C12—C17—H17	118.9	C30—C35—H35	118.6
C16—C17—H17	118.9	C34—C35—H35	118.6
O3—C18—H18A	109.5	O6—C36—H36A	109.5
O3—C18—H18B	109.5	O6—C36—H36B	109.5
H18A—C18—H18B	109.5	H36A—C36—H36B	109.5
O3—C18—H18C	109.5	O6—C36—H36C	109.5
H18A—C18—H18C	109.5	H36A—C36—H36C	109.5
H18B—C18—H18C	109.5	H36B—C36—H36C	109.5
			
C1—N2—N3—C2	1.75 (19)	C19—N5—N6—C20	−0.04 (18)
N3—N2—C1—N1	−0.14 (17)	N6—N5—C19—N4	−0.11 (18)
N3—N2—C1—C10	−179.76 (14)	N6—N5—C19—C28	−178.32 (15)
C2—N1—C1—N2	−1.41 (18)	C20—N4—C19—N5	0.22 (18)
C3—N1—C1—N2	−176.04 (14)	C21—N4—C19—N5	174.33 (14)
C2—N1—C1—C10	178.19 (15)	C20—N4—C19—C28	178.48 (14)
C3—N1—C1—C10	3.6 (3)	C21—N4—C19—C28	−7.4 (2)
N2—N3—C2—O1	176.86 (16)	N5—N6—C20—O4	−179.34 (16)
N2—N3—C2—N1	−2.54 (18)	N5—N6—C20—N4	0.17 (18)
C1—N1—C2—O1	−177.09 (17)	C19—N4—C20—O4	179.31 (16)
C3—N1—C2—O1	−2.1 (3)	C21—N4—C20—O4	4.8 (3)
C1—N1—C2—N3	2.33 (17)	C19—N4—C20—N6	−0.23 (16)
C3—N1—C2—N3	177.33 (13)	C21—N4—C20—N6	−174.72 (13)
C1—N1—C3—C8	−125.99 (18)	C19—N4—C21—C26	121.19 (18)
C2—N1—C3—C8	60.1 (2)	C20—N4—C21—C26	−65.6 (2)
C1—N1—C3—C4	56.1 (2)	C19—N4—C21—C22	−61.6 (2)
C2—N1—C3—C4	−117.72 (18)	C20—N4—C21—C22	111.66 (18)
C8—C3—C4—C5	1.4 (2)	C26—C21—C22—C23	0.5 (2)
N1—C3—C4—C5	179.21 (14)	N4—C21—C22—C23	−176.62 (14)
C9—O2—C5—C4	6.1 (2)	C27—O5—C23—C22	−5.2 (2)
C9—O2—C5—C6	−172.28 (15)	C27—O5—C23—C24	172.59 (15)
C3—C4—C5—O2	−179.94 (15)	C21—C22—C23—O5	177.57 (15)
C3—C4—C5—C6	−1.6 (2)	C21—C22—C23—C24	−0.1 (2)
O2—C5—C6—C7	179.35 (15)	O5—C23—C24—C25	−178.55 (15)
C4—C5—C6—C7	0.9 (3)	C22—C23—C24—C25	−0.7 (3)
C5—C6—C7—C8	0.1 (3)	C23—C24—C25—C26	1.1 (3)
C4—C3—C8—C7	−0.4 (2)	C24—C25—C26—C21	−0.7 (3)
N1—C3—C8—C7	−178.24 (15)	C22—C21—C26—C25	−0.1 (3)
C6—C7—C8—C3	−0.3 (3)	N4—C21—C26—C25	176.97 (15)
N2—C1—C10—C11	−104.32 (18)	N5—C19—C28—C29	5.5 (2)
N1—C1—C10—C11	76.1 (2)	N4—C19—C28—C29	−172.56 (15)
C1—C10—C11—C12	167.77 (14)	C19—C28—C29—C30	−173.76 (14)
C10—C11—C12—C13	103.80 (17)	C28—C29—C30—C31	−19.9 (2)
C10—C11—C12—C17	−73.44 (18)	C28—C29—C30—C35	160.65 (15)
C17—C12—C13—C14	0.6 (2)	C35—C30—C31—C32	−0.6 (2)
C11—C12—C13—C14	−176.69 (15)	C29—C30—C31—C32	179.94 (15)
C12—C13—C14—C15	−1.3 (2)	C30—C31—C32—C33	1.8 (2)
C18—O3—C15—C16	−2.5 (2)	C36—O6—C33—C34	−1.8 (2)
C18—O3—C15—C14	176.08 (13)	C36—O6—C33—C32	178.02 (14)
C13—C14—C15—O3	−177.57 (14)	C31—C32—C33—C34	−1.8 (2)
C13—C14—C15—C16	1.1 (2)	C31—C32—C33—O6	178.34 (14)
O3—C15—C16—C17	178.24 (15)	O6—C33—C34—C35	−179.42 (14)
C14—C15—C16—C17	−0.3 (2)	C32—C33—C34—C35	0.8 (2)
C13—C12—C17—C16	0.2 (2)	C31—C30—C35—C34	−0.5 (2)
C11—C12—C17—C16	177.54 (15)	C29—C30—C35—C34	179.00 (15)
C15—C16—C17—C12	−0.3 (2)	C33—C34—C35—C30	0.4 (2)

### Hydrogen-bond geometry (Å, °)

**Table:**

*D*—H···*A*	*D*—H	H···*A*	*D*···*A*	*D*—H···*A*
N3—H3N···O4^i^	0.972 (18)	1.784 (19)	2.7463 (18)	169.6 (17)
N6—H6N···O1^ii^	0.931 (16)	1.936 (17)	2.8429 (18)	164.2 (16)

Symmetry codes: (i) −*x*+1, *y*+3/2, −*z*+1/2; (ii) −*x*+1, *y*−3/2, −*z*+1/2.
